# Concurrent mood pathology in patients with autism spectrum disorder (ASD). A case report.

**DOI:** 10.1192/j.eurpsy.2024.786

**Published:** 2024-08-27

**Authors:** C. Díaz Mayoral, M. Martín de Argila Lorente, E. Arroyo Sánchez, P. Setién Preciados

**Affiliations:** ^1^Psiquiatría, Hospital Universitario Príncipe de Asturias; ^2^Psiquiatría, Hospital Doctor Rodríguez Lafora, Madrid, Spain

## Abstract

**Introduction:**

Mood disorders in patients with ASD (Autism Spectrum Disorder) have a significant impact on their well-being. Major depression and bipolar disorder are among the most common co-occurring psychiatric diagnoses in autism. Prevalence estimates range from 10-50% for depression and approximately 5% for bipolar disorder. These figures are markedly higher than those reported in the general population.

The diagnosis of these disorders in patients with autism poses several challenges: mood problems may be “overshadowed” by the diagnosis itself, symptoms vary between individuals and may present “atypically” (psychomotor agitation, regression, reduced self-care, and severe irritability). The use of assessment tools based largely on criteria developed and validated in the general population is common.

**Objectives:**

A case of a patient diagnosed with ASD and co-occurring mood disorder is presented followed by a theoretical review on the topic.

**Methods:**

A case is presented with a bibliographic review.

**Results:**

A 20-year-old patient with a diagnosis of severe autism spectrum disorder was referred to the emergency department for behavioral disturbances based on episodes of heteroaggressiveness and self-aggressiveness, with a daily frequency, in the last 2 months. His parents attribute this decompensation to the introduction of Sertraline and changes in his routine, which has implied less stimulation. Having ruled out underlying organic pathology, given that her father refers to frequent episodes of crying and abandonment of leisure activities of his liking, we suspect a mood disorder.

In hospitalization, Sertraline was withdrawn and Valproic Acid was introduced. Likewise, Risperidone dose was increased, already prescribed in outpatient care. Progressively, a notable improvement was observed.

**Conclusions:**

Current clinical recommendations on the use of selective serotonin reuptake inhibitors (SSRIs) for mood problems are largely based on evidence from typically developing groups. However, it has been shown that some individuals with autism show different neural responses to pharmacological challenge compared to neurotypical individuals. In addition, the use of SSRIs in ASD may result in increased adverse side effects, such as agitation, impulsivity, hyperactivity, stereotypy, and insomnia, and it has been suggested that they should therefore only be considered on a “case-by-case” basis. A systematic review reported that mood stabilizers (Lithium, Valproic Acid) are preferable to atypical antipsychotics, which are associated with a large number of side effects.

Because of the lack of strong evidence on the efficacy of pharmacologic interventions and issues regarding safety and side effects, risperidone and aripiprazole are among the few medications approved by the FDA for the treatment of irritability in people with autism. More research aimed at effective medications to treat mood problems in ASD needs to be advocated.

**Disclosure of Interest:**

None Declared

